# Associated Clinical Factors for Coagulation Dysfunction due to *Trimeresurus stejnegeri*: A Retrospective Observational Study

**DOI:** 10.1155/2023/8832355

**Published:** 2023-04-30

**Authors:** Run-Hua Xie, Xiao-Lu Ye, Cong-Yao Tang, Yu-Huai Wang, Long-Xin Zhong

**Affiliations:** Shenzhen Hospital, Beijing University of Chinese Medicine, Shenzhen, China

## Abstract

**Background:**

*Trimeresurus stejnegeri* (T.s) accounts for most snakebites in southern China, which always leads to coagulation dysfunction. Coagulopathy due to venom is widely considered to be a characteristic phenomenon of the DIC-like syndrome. It is vitally important for first-line clinicians to improve this condition as soon as possible. However, clinical factors associated with coagulation function in *Trimeresurus stejnegeri* has not been well characterized yet.

**Materials:**

Patients bitten by vipers were admitted to the Emergency Department of a hospital in Shenzhen, southern China, from 2021 to 2022 and were retrospectively reviewed. Patient clinical characteristics and laboratory data were compared in the eligible patients bitten by T.s by their prothrombin time (PT), fibrinogen level (FIB), and platelet count on 2-3 days after bitten.

**Results:**

105 patients were included in this study. The mean values of PT, FIB, and PLT are as follows: 12.8 ± 0.79 s, 2.25 ± 0.47 g/L, and 196.2 ± 57.1 × 10^9^/L. Uric acid (UA) (367.9 ± 103.85), blood glucose (6.53 + 1.64) show negative trend of correlation, while CRP (2.12 + 4.17) shows positive trend of association with coagulation function. The smoke and systolic blood pressure may exert negative effects on PT and PLT, respectively. Logistic regression analysis indicated that uric acid (UA) shows significant connection with PT (OR = 1.15 and *P* value <0.0001), FIB (OR = 0.89 and *P* value = 0.026), and PLT (OR = 0.79 and *P* value = 0.007). CRP is also shown to be associated with FIB (OR = 1.33 and *P* value = 0.043).

**Conclusion:**

: Uric acid (UA) shows a significant association with PT, FIB, and PLT. CRP is related to FIB. Blood glucose shows a negative trend of correlation with PT. We do recommend physician should low the level of UA in some degree on the basis of injection of an antivenom serum.

## 1. Background

Snakebite remains a very common medical emergency in China [1]. *Trimeresurus stejnegeri* (T.s), also known locally as the bamboo snake, accounts for most human envenomation cases in south China, such as Guang-Dong, Tai-Wan, Guang-Xi, and San-Ya [2–6]. The ratio of T.s bitten ranks high among the dozes of venomous snake bites in China. According to a ten-year retrospective review of 1,107 snakebite patients, the bite of T.s accounts for 46.2% [5].

The T.s bite always induces tissue swelling, pain, and, more commonly, subcutaneous hemorrhagic ecchymosis and visceral hemorrhage due to varying degrees of coagulopathy. However, T.s venom is relatively moderate compared with most venoms due to its lethal dose is 100 mg while the amount of single T.s bite is only 5.1 mg [7]. Composition of T.s venoms is complex which mainly contains protease, hemorrhagic toxins, phospholipase, thrombin-like enzyme, and multiple promoting coagulation factors [8–10]. The venom protease can directly or indirectly acts on the blood vessel wall, destroying the relevant structure of the blood vessel wall and inducing the release of bradykinin, histamine, and 5-hydroxytryptamine. The releasing factors can destroy the capillary endothelial cells and inhibits platelet aggregation, leading to bleeding. The venom hemorrhagic toxins can directly act on blood cell membrane to increase its permeability and brittleness. Phospholipase A2 can make the blood lecithin hydrolyzed to lysate lecithin, producing hemolysis. Promoting coagulation factor of T.s venom produce a thrombin-like enzymes effect which is responsible for defibrination syndrome as well as hyperfibrinogenemia [11], considering to be a characteristic phenomenon of the DIC-like syndrome, which can bring potentially life-threatening bleeding. At the same time, venom kinetics revealed that the activity maintenance of T.s venoms can keep for two weeks, which was associated with prolonged coagulopathy [6, 7, 12]. In general, the mechanism of T.s venoms is to achieve coagulation promotion and anticoagulation through the abnormal consumption of fibrinogen, coagulation factors and platelets, induction or inhibition of platelet aggregation, and dyfunction of coagulation factors [13]. As a result, the coagulation function test will often demonstrate as APTT, PT, TT prolongs, and Fib decrease.

Subcutaneous hemorrhagic ecchymosis and visceral hemorrhage due to varying degrees of coagulopathy are the main clinical signs of T.s bite. However, for limited localised signs caused by a small quantity of venom, it often appears as a tiny hemorrhagic spot with pain and tissue swelling. While for more severe coagulopathy, which is collectively referred to as venom-induced consumption coagulopathy (VICC), which is manifested as bleeding, minor subcutaneous bleeding, nose bleeding, and gingival bleeding, in severe cases, it can cause a state of blood coagulation, persistent bleeding from the wound, hematuria, gastrointestinal bleeding, and even cerebral hemorrhage [14]. Due to microembolisation or thrombo-embolism in the early or late phases, procoagulative clinical complications may also occur, resulting in DIC-like consumption disorders, and finally, to organ dysfunction syndrome [15].

Fortunately, death due to T.s bite is rare. Antivenom remains the most effective treatment to improve the coagulation function and should be administrated as soon as possible; the recommended antivenom for T.s bite is Agkistrodon Acutus Antivenin. According to the package insert, Agkistrodon Acutus Antivenin come from equine immunoglobulin against pentaphylla venom after digestion by gastric enzymes and stores at 2–8°C, the initial dose for intravenous injection is usually 4000 u–8000 u. The addition dose depends on the severity of the disease, but a single large dose of antivenom may increase the risk of allergy or serological disease. Usually, the median time for recovery of coagulation function is 6 hours after the use of neutralizing dose of antivenin [16]. Therefore, it is recommended to perform clinical and laboratory tests every 6–8 hours. Based on the test results, 4000 U can be considered each time. The additional therapy often takes at least 2-3 times. Agkistrodon Hyalys Antivenin is also recommended to add with Agkistrodon Acutus Antivenin if necessary. Besides, some researchers recommended using clotting factor replacement. But the improvement of coagulation dysfunction is sometimes unsatisfactory after antivenom administration as well as clotting factor replacement [17, 18]. Many researchers have reported that amounts of clinical characteristic show association with coagulation function. For example, the smoked patients are always in a hypercoagulable state [19–22]. While in the treatment of T.s bite, these clinical characteristics seem important but are often neglected due to objective reasons such as patient's compliance and economic cost, and more likely, the lack of understanding and uncertainty of its value. Hence, given the lack of evidence on the effects of factors such as BMI, smoke, and blood pressure generating on recovery of coagulation function, this retrospective observational study aims to investigate the clinical materials of T.s victims after antivenom administration and dig out valuable information.

## 2. Methods

Informed consent was obtained from the patient(s) for their anonymized information to be published in this article.

**Figure 1:**
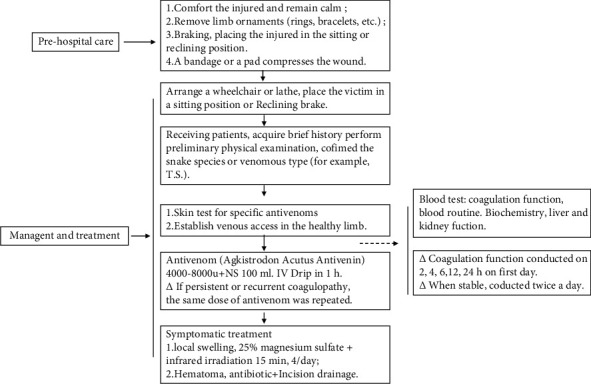
Flowchart of the clinical management of T.s bite.

In this study, we utilize the diagnostic criteria for venomous snakebite of *Trimeresurus stejnegeri* for reference: (1) the responsible venomous snake should be confirmed as T.s; (2) the appearance and morphology of the venomous snake described by the patient basically conformed to the characteristics of T.s; (3) clinical manifestations of hepatotoxin included local swelling, severe wound pain, and subcutaneous ecchymosis in some patients; having (1) or both (2) and (3) could be diagnosed; (4) pregnant women and children under 10 years old were excluded; (5) Length of hospital stay >24 h. Therapeutic management after *Trimeresurus* bite including laboratory examination especially coagulation function tests at 2, 4, 6, 12, and 24 h on first day. When stable, coagulation function test will be conducted twice a day. Treatment for snakebites included antivenom, antitetanus, steroids, antibiotics, and clotting factor replacement therapy. The recommended initial dose of was one vial (8000 u) which is expected to neutralize the average venom yield. The same dose was repeated six hours after the initial dose of antivenom owing to persistent or recurrent coagulopathy [23]. The detailed clinical management of a *Trimeresurus steinjeri* bite is showed, as a flowchart in Figure 1.

**Figure 2:**
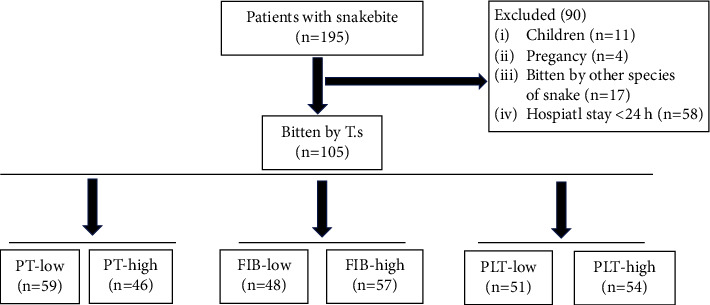
Study flowchart.

We retrospectively reviewed patients of venomous snakebite admitted to Emergency Department of Shenzhen hospital, Beijing University of Chinese, between 2021 and 2022, all objective clinical parameters were recorded using a preformatted clinical data form that included sociodemographic data, snake species, epidemiological data, and various pertinent laboratory results such as BMI, smoke, systolic and diastolic blood pressure, complete blood count (CBC), serum potassium, blood urea nitrogen (BUN), uric acid (UA), creatinine, APTT, PT, FIB, and D-dimer. Prothrombin time (PT) is reported to be effective and sensitive for the assessment of viper-bitten patients, recommended to be used as an indication of coagulation function for antivenom administration [12]. Fibrinogen (FIB) level is considered to be worthy to estimate if the coagulopathy has been resolved. Platelet (PLT) is another factor cannot be ignored that maintain the well-balanced coagulation function. The cut-off value of PT, FIB, and PLT are the mean value of 105 eligible T.s victims tested on 2-3 days after bitten, respectively. The PT-low and PT-high groups were defined as PT ≤ 12.87 s and PT > 12.87 s. The FIB-low and FIB-high groups were also defined as FIB ≤ 2.25 s and FIB > 2.25 s. The PLT-low and PLT-high groups were defined as PLT ≤ 196.2 *∗* 10^9^/L and PLT > 196.2 *∗* 10^9^/L (Figure 2).

Continuous data were showed as mean ± standard deviations (mean ± SD). To identify the potential risk factors associated with PT, FIB, and PLT variations, the distinct groups were compared using the independent sample *t*-test or the Mann–Whitney *U* test. Categorical data were presented as percentages or frequencies and between groups comparison used the chi-square or Fisher exact test. Factors significantly connected with PT, FIB, and PLT variations were put in both logistic regression presents; odds ratios (ORs) as well as relevant 95% confidence intervals (CIs) were reported. The data were analyzed utilizing Prism 9 (GraphPad, La Jolla, CA). A two-tailed *P* value <0.05 was considered statistically significant.

## 3. Results

### 3.1. Coagulation Function Related to 105 Patients on 2-3 Days after Bitten by T.s

After 105 patients accepted the similar treatment scheme, we collected and analyzed the results of coagulation function on 2-3 days after being bitten by T.s. As is shown in Table 1, the mean values of PT, FIB, and PLT are as follows: 12.8 ± 0.79 s, 2.25 ± 0.47 g/L, 196.2 ± 57.1 × 10^9^/L, which were used as cut-off values to help to define the PT-low (≤12.8 s) group and the PT-high (>12.8 s) group, the FIB-low (≤2.25 g/L) group and the PT-high(>2.25 g/L) group, the PLT-low (≤196.2 × 10^9^/L) group, and the PLT-high (>196.2 × 10^9^/L) group.

### 3.2. Characteristic Data of the Patients Grouped According to PT, FIB, and PLT

As shown in Table 2, the baseline characteristics of the victims were well matched between the 2 groups, for the PT-low and PT-high groups show no significant difference in the sex proportion, age, BMI, time to enter hospital, time after bitten to first antivenom dose, pain score, circumference of swollen, systolic and diastolic blood pressure, swollen degrees, and most laboratory tests such as leukocyte. Of note, compared with the PT-high group, the proportion of smoked in the PT-low group significantly decreased (11.3% vs. 39.3% and *P*=0.022), platelet count decreased (214.8 ± 66.8 vs. 243.3 ± 55.8 and *P*=0.03), uric acid (UA) decreased (298.6 ± 72.4 vs. 456.9 ± 65.2 and *P* < 0.0001), blood glucose decreased (6.25 ± 1.1.7 vs. 6.92 ± 1.48 and *P*=0.047), initial PT decreased (12.5 ± 1.14 vs. 17.5 ± 25.2 and *P*=0.001), INR increased (0.934 ± 0.12 vs. 1.117 ± 0.64 and *P*=0.006), and PTA decreased (117 ± 16.3 vs. 99.47 ± 22.7 and *P* < 0.0001).

As demonstrated in Table 3, the baseline characteristics including sex proportion, age, circumference of swollen, and so on of the FIB-low and FIB-high groups showed no significant difference. Compared with FIB-high group, systolic blood pressure increased (133.1 ± 20.3 vs. 83.1 ± 14.4 and *P* < 0.0001), CRP decreased (1.237 + 0.98 vs. 2.172 + 6.09 and *P*=0.036), uric acid (UA) increased (399.3 ± 97.8 vs. 340.5.9 ± 106.9 and *P*=0.003), initial PTA decreased (103.1 ± 23.5 vs. 114.3 ± 17.8 and *P*=0.007), and FIB decreased (2.42 ± 1.57 vs. 3.03 ± 0.54 and *P*=0.007).

In Table 4, the initial characteristics of T.s bite victims grouped by PLT on 2-3 days showed no significant difference in the sex proportion, age, BMI, time to enter hospital, time after bitten to first antivenom dose, pain score, circumference of swollen, systolic and diastolic blood pressure, swollen degrees and most laboratory tests such as leukocyte. Compared with PLT-high group, initial platelet in PLT-low count decreased (260.0 ± 59.7 vs. 197.4 ± 41.4 and *P* < 0.0001), and UA increased (400.7 + 102.8 vs. 335.8 + 94.5 and *P*=0.003).

**Figure 3:**
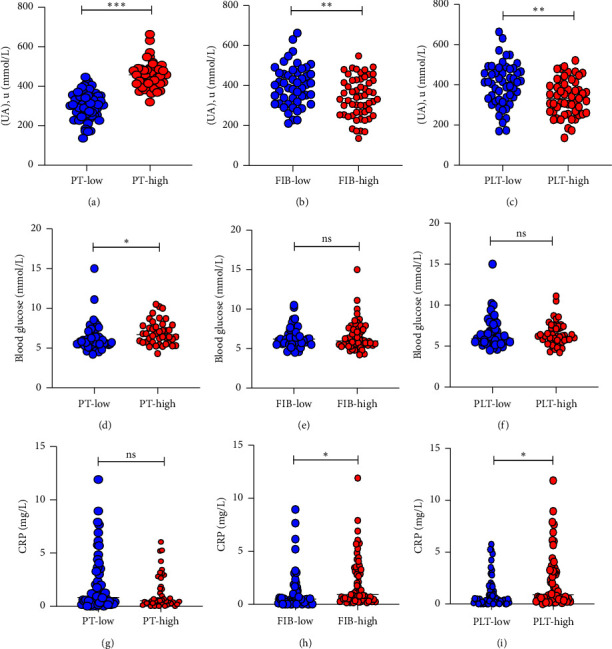
Comparison of uric acid (UA) (a–c), blood glucose (d–f), CRP (g–i) in T.s bite victims grouped by PT, FIB, and PLT (ns, no significance; ^*∗*^*P* < 0.05; ^*∗∗*^*P* < 0.01; ^*∗∗∗*^*P* < 0.001).

On the whole, more evidence showed that uric acid (UA) (Figures 3(a)–3(c)), blood glucose (Figures 3(d)–3(f)) seem to shows a negative correlation, while CRP (Figures 3(g)–3(i)) shows a positive association with coagulation function. The smoke and systolic blood pressure also exert negative effects on PT and PLT, respectively.

### 3.3. Identify the Factors Associated with Coagulation Function of T.s Bite Victims

**Figure 4:**
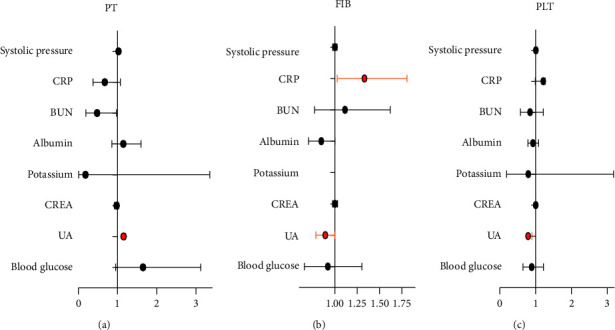
Logistic regression analyses of associated factors for in 105 T.s bite victims.

- To further identify the factors associated with PT, FIB, and PLT, 105 cases of T. s bite victims were put in the multiple logistic regression analysis. As is demonstrated in Table 5 and Figure 4, uric acid (UA) shows significant connection with PT (OR = 1.15 and *P* value <0.0001), FIB (OR = 0.89 and *P* value = 0.026), and PLT (OR = 0.79 and *P* value = 0.007). CRP is also shown to be associated with FIB (OR = 1.33 and *P* value = 0.043).

## 4. Discussion

It is well accepted that patients bitten by blood-toxic venomous snakes would suffer different kinds of blood coagulation disorder. Though there are fewer reports on DIC evoked by snakebites, but the DIC- like syndrome, more frequently, could be caused by amounts of snake especially T.s in south China [13, 26]. Once admittedto hospital and diagnosed, the dominating strategy is antivenom as well as combination with other treatment, which can reduce the occurrence of secondary/long term complications [27]. Even so, antivenom is considered not completely to make coagulopathy right invariety of snakebites from venomous species. Antivenom can just work on the unbound free venom in circulation, while the entered venom has already resulted in a cascade of coagulation factor consumption. Many physicians sought mounts of methods to replenish the lack of antivenom effectiveness on coagulopathy status post snakebites, but the effectiveness remains controversial and is still investigation [2, 28–30].

Coagulation disorders by snakebites always not only prolong the length of hospitalization, but also aggravate limb swelling and inflammation, which means hard nut to crack for first-line clinician. It is reported that procoagulant, lectins, and other coagulation toxins in venom of *Trimeresurus stejnegeri* (T.s) can result in abnormal coagulation function, longer prothrombin time, and lower fibrinogen [31–33]. In clinical work, it is a very common phenomenon that T.s victims' shows great difference in recovery of coagulation function after basically similar treatment. It is difficult to get past the hypothesis that some clinical characteristics of bite victims may exert significant impact on improvement of coagulation function. Basic features such as age [34], BMI [34], smoke [35], blood pressure [36], and so on shows close association with coagulation functions. Many scholars also provide mounts of evidences that blood test such as glucose fluctuation will generate strong impact on coagulation function [37]. While in the treatment of T.s bite, these characteristics seems important but are often get too little attention due to more importantly, the lack of understanding of clinical characteristic. Here, we analyzed the characteristic data, clinical manifestations, and laboratory findings of 105 patients. Blood glucose (6.53 + 1.64) seems to shows negatively correlation, while CRP (2.12 + 4.17) shows a positive association with coagulation function. The smoke and systolic blood pressure also exert negative effect on PT and PLT, respectively. Surprisingly, the result indicates that uric acid (UA) was positively related to prolongation of PT, while positively related to prolongation of FIB and PLT count. In the other word, the elevation of UA within limits (367.9 ± 103.85 *μ*mmol/L) may restraint the recovery of coagulation function in T.s victim. Most previously reported data suggested that high and continuous level of UA, hyperuricemia, defined as its concentrations higher than 405 mmol/L (6.8 mg/dL) as this is urate's solubility point measured using automated enzymatic methods in laboratories [38]. Some scholars insisted that UA, as a fuse inducing oxidative stress and inflammation will promote damage to vascular endothelium and activate the coagulation system, putting the body in a hypercoagulable state [39–41]. However, Petreski et al. [42] reported that UA may a have a J-shaped relationship with all-cause mortality, meaning that it is not just positive regulation or negative regulation of UA affecting homeostasis. Our finding is similar to the small part of reported data. Some researchers believed that low uric acid may act as effective oxidant that can enhances thrombolysis, potentially by inhibiting oxidative stress, which prevents fibrinolysis by alteplase in thrombi [21, 38, 43]. At this point, considering more and more people suffer from hyperuricemia at the same time, we do recommend physician should low the level of UA in some degree on the basis of injection of antivenom serum.

## 5. Limitations

This study was an observational retrospective review; it is inevitable the selection bias exists. The small sample size is another limitation. Our findings should be evaluated in a properly designed prospective study, the investigation about accurate and appropriate levels of UA, CRP, and blood glucose should be carried out in the future. Since the degree of coagulopathy may vary among snake species, the results from T.s should not be generalized to other species of venomous snakes unless getting enough validation.

## 6. Conclusion

Uric acid (UA) shows a significant association with PT, FIB, and PLT. CRP is positively related to FIB. Blood glucose shows a negative trend of correlation with PT. We do recommend physician should low the level of UA in some degree on the basis of injection of antivenom serum.

## Figures and Tables

**Table 1:** Follow up characteristics of 105 patients on 2-3 days after bitten.

Characteristics	Mean values	Standard deviation
Clinical manifestations		
Pain score	0.78	0.11
Circumference of swollen limb (cm)	0.53	0.09
Laboratory test		
Platelets (×10^9^/L)	196.2	57.1
Prothrombin time (PT) (second)	12.8	0.79
International standard ratio of prothrombin (INR) (%)	0.97	0.08
Prothrombin activity (PTA) (%)	108.0	14.1
Activated partial thromboplastin time (second)	32.8	3.83
Fibrinogen (FIB) (g/L)	2.25	0.47
Thrombin time (TT) (second)	18.0	1.39
D-dimer, D-Di (*μ*g/ml)	2.45	3.40

**Table 2:** Basal characteristics of 105 patients when first admitted to hospital classified by prothrombin time on 2-3 day.

Characteristics	PT-low (*n* = 59)	PT-high (*n* = 46)	*P* values
Personal factors			
Male, *n* (%)	34 (57.6)	29 (63.0)	0.689
Age (years) ($\bar{x}$ ± *s*)	43.3 ± 16.3	37.5 ± 12.7	0.054
BMI (kg/m^2^) ($\bar{x}$ ± *s*)	21.3 ± 7.35	22.0 ± 7.02	0.805
Smoke (%)	6 (11.3)	13 (39.3)	0.022^*∗*^
Systolic blood pressure ($\bar{x}$ ± *s*)	139.5 ± 22.6	133.0 ± 18.9	0.401
Diastolic blood pressure ($\bar{x}$ ± *s*)	84.2 ± 15.2	80.8 ± 11.7	0.224
Comorbidity^&^			
Chronic lung disease, *n* (%)	2 (3.38)	1 (2.17)	0.711
Chronic renal failure, *n* (%)	0 (0.00)	0 (0.00)	—
Hepatic dysfunction, *n* (%)	1 (1.69)	0 (0.00)	0.375
Cardiovascular diseases, *n* (%)	2 (3.38)	3 (6.50)	0.455
Hematologic diseases, *n* (%)	0 (0.00)	0 (0.00)	—
Clinical manifestations			
Time to enter hospital (h)	2.74 ± 3.78	1.93 ± 1.74	0.704
Pain score ($\bar{x}$ ± *s*)	1.77 ± 0.55	2.00 ± 1.20	0.054
Circumference (cm) ($\bar{x}$ ± *s*)	2.01 ± 1.26	1.75 ± 0.73	0.596
Swollen degree^#^	Mild (45), Moderate (14)	Mild (35), Moderate (11)	>0.99
Time to first antivenom dose (h)	3.55 ± 3.59	2.68 ± 0.89	0.407
Laboratory test			
Leukocyte count (10^9^/L)	7.99 ± 2.75	9.03 ± 4.69	0.111
Neutrophil count (%)	62.1 ± 12.94	63.5 ± 13.31	0.355
Erythrocyte count (10^12^/L)	4.89 + 0.47	4.98 + 0.73	0.401
Hemoglobin (g/L)	141.3 ± 15.9	143.7 ± 15.5	0.572
Platelet (10^9^/L)	243.3 ± 55.8	214.8 ± 66.8	0.030^*∗*^
CRP (mg/L)	2.08 ± 6.15	1.29 ± 1.59	0.085
Albumin (g/L)	44.1 ± 3.13	44.1 ± 3.59	0.307
Urea (BUN) (mmol/L)	5.32 ± 1.43	4.98 ± 1.31	0.578
Potassium (mmol/L)	3.63 ± 0.32	3.52 ± 0.32	0.706
Creatinine (CREA) (*μ*mmol/L)	64.17 ± 20.4	64.8 ± 17.9	0.710
Uric acid (UA) (*μ*mmol/L)	298.6 ± 72	4 456.9 ± 65.2	<0.0001^*∗∗∗*^
Blood glucose (mmol/L)	6.25 ± 1.71	6.92 ± 1.48	0.047^*∗*^
Prothrombin time (PT) (second)	12.5 ± 1.14	17.5 ± 25.2	0.001^*∗∗*^
International standard ratio of prothrombin-(INR)	0.934 ± 0.12	1.117 ± 0.64	0.006^*∗∗*^
Prothrombin activity (PTA) (%)	117 ± 16.3	99.47 ± 22.7	<0.0001^*∗∗∗*^
APTT (s)	37.3 ± 6.84	40.4 ± 13.5	0.148
Fibrinogen (FIB) (g/L)	2.86 ± 0.57	5.46 ± 0.72	0.217
Thrombin time (TT) (s)	16.79 ± 1.51	24.6 ± 37.3	0.108

^#^Swollen degree was estimated by Blaylock classification [24]. ^&^Chronic lung diseases refer to chronic obstructive pulmonary disease (COPD) bronchiectasis mainly; chronic renal failure is diagnosed by physician based on Clinical Practice Guideline for Diabetes and CKD (KDIGO): 2012 update [25]; hepatic dysfunction refer to acute and chronic hepatitis of various etiologies; cardiovascular diseases including coronary heart disease, cor pulmonale; hematologic diseases including hemophilia, anemia.

**Table 3:** Basal characteristics of 105 patients when first admitted to hospital classified by fibrinogen (FIB) on 2-3 day.

Characteristics	FIB-low (*n* = 48)	FIB-high (*n* = 57)	*P* values
Personal factors			
Male, *n* (%)	34 (58.6)	30 (52.6)	0.072
Age, years ($\bar{x}$ ± *s*)	38.8 ± 14.7	42.4 ± 15.3	0.225
BMI (kg/m^2^) ($\bar{x}$ ± *s*)	23.7 ± 3.57	23.5 ± 3.71	0.827
Smoke, *n* (%)	12 (33.3)	10 (21.2)	0.471
Systolic blood pressure ($\bar{x}$ ± *s*)	133.1 ± 20.3	83.1 ± 14.4	<0.0001^*∗∗∗*^
Diastolic blood pressure ($\bar{x}$ ± *s*)	83.0 ± 13.2	82.6 ± 14.4	0.727
Comorbidiary^&^			
Chronic lung disease, *n* (%)	1 (2.08)	2 (3.51)	0.662
Chronic renal failure, *n* (%)	0 (0.00)	0 (0.00)	—
Hepatic dysfunction, *n* (%)	1 (2.08)	0 (0.00)	0.274
Cardiovascular diseases, *n* (%)	2 (4.16)	3 (5.26)	0.793
Hematologic diseases, *n* (%)	0 (0.00)	0 (0.00)	—
Clinical manifestations			
Time to enter hospital (h)	3.17 ± 4.07	2.74 ± 3.94	0.581
Pain score ($\bar{x}$ ± *s*)	1.92 ± 0.64	1.84 ± 0.65	0.558
Circumference (cm) ($\bar{x}$ ± *s*)	1.86 ± 0.98	1.88 ± 1.29	0.931
Swollen degree^#^	Mild (35) Moderate (17)	Mild (44) Moderate (8)	0.065
Time to first antivenom dose (h)	3.50 ± 3.49	3.25 ± 2.97	0.693
Laboratory test			
Leukocyte count (10^9^/L)	8.67 ± 4.49	8.26 ± 3.00	0.571
Neutrophil count (%)	61.1 ± 13.21	63.7 ± 12.98	0.418
Erythrocyte count (10^12^/L)	4.99 ± 0.56	4.89 ± 0.64	0.386
Hemoglobin (g/L)	144.8 ± 15.3	140.3 ± 15.9	0.149
Platelet (10^9^/L)	221.3 ± 53.3	238.9 ± 68.3	0.149
CRP (mg/L)	1.237 ± 0.98	2.172 ± 6.09	0.036^*∗*^
Albumin (g/L)	43.9 ± 2.76	43.8 ± 3.46	0.793
Urea (BUN) (mmol/L)	5.11 ± 1.48	5.22 ± 1.31	0.705
Potassium (mmol/L)	3.52 ± 0.32	3.64 ± 0.32	0.052
Creatinine (CREA) (*μ*mmol/L)	67.3 ± 18.4	62.8 ± 19.8	0.237
Uric acid (UA) (*μ*mmol/L)	399.3 ± 97.8	340.5 ± 106.9	0.003^*∗∗*^
Blood glucose (mmol/L)	6.49 ± 2.37	6.58 ± 1.82	0.797
Prothrombin time (PT) (second)	22.8 ± 2.34	12.7 ± 1.23	0.049^*∗*^
International standard ratio of prothrombin-(INR)	1.003 ± 0.21	0.959v0.13	0.115
Prothrombin activity (PTA) (%)	103.1 ± 23.5	114.3 ± 17.8	0.007^*∗∗*^
APTT (s)	42.6 ± 13.5	37.8 ± 6.99	0.152
Fibrinogen (FIB) (g/L)	2.42 ± 1.57	3.03 ± 0.54	0.007^*∗∗*^
Thrombin time (TT) (s)	29.23 ± 47.9	19.16 ± 20.5	0.153

^#^Swollen degree was estimated by Blaylock classification [24]. ^&^Chronic lung diseases refer to chronic obstructive pulmonary disease (COPD) bronchiectasis mainly; chronic renal failure is diagnosed by physician based on Clinical Practice Guideline for Diabetes and CKD (KDIGO): 2012 update [25]; hepatic dysfunction refer to acute and chronic hepatitis of various etiologies; cardiovascular diseases including coronary heart disease, cor pulmonale; hematologic diseases including hemophilia, anemia.

**Table 4:** Characteristic data, clinical manifestations, and laboratory findings of 105 patients when first admitted to hospital classified by Platelet on 2-3 day.

Characteristics	PLT-low (*n* = 52)	PLT-high (*n* = 53)	*P* values
Personal factors			
Male, *n* (%)	32 (61.5)	34 (62.9)	>0.99
Age, years ($\bar{x}$ ± *s*)	40.9 ± 13.9	40.2 ± 16.2	0.818
BMI, (kg/m^2^) ($\bar{x}$ ± *s*)	23.7 ± 3.43	22.2 ± 5.67	0.104
Smoke (%)	9 (17.3)	10 (18.8)	0.836
Systolic blood pressure ($\bar{x}$ ± *s*)	132.3 ± 22.8	139.0 ± 24.1	0.153
Diastolic blood pressure ($\bar{x}$ ± *s*)	83.0 ± 11.1	82.6 ± 16.2	0.877
Comorbidity^&^			
Chronic lung disease, *n* (%)	1 (1.92)	2 (3.77)	0.569
Chronic renal failure, *n* (%)	0 (0.00)	0 (0.00)	—
Hepatic dysfunction, *n* (%)	1 (1.92)	0 (0.00)	0.310
Cardiovascular diseases, *n* (%)	3 (5.76)	2 (3.77)	0.631
Hematologic diseases, *n* (%)	0 (0.00)	0 (0.00)	—
Clinical manifestations			
Time to enter hospital (h)	2.42 ± 3.20	3.29 ± 4.49	0.252
Pain score ($\bar{x}$ ± *s*)	1.98 ± 0.71	1.78 ± 0.57	0.133
Circumference (cm) ($\bar{x}$ ± *s*)	1.86 ± 1.26	1.88 ± 1.15	0.931
Swollen degree^#^	Mild (35)	Mild (44)	0.065
	Moderate (17)	Moderate (8)	
Time to first antivenom dose (h)	3.20 ± 2.89	3.51 + 3.55	0.629
Laboratory test			
Leukocyte count (10^9^/L)	8.11 ± 4.49	8.84 ± 2.90	0.335
Neutrophil count (%)	61.6 ± 13.49	63.4 ± 12.59	0.492
Erythrocyte count (10^12^/L)	4.90 ± 0.55	4.98 ± 0.65	0.463
Hemoglobin (g/L)	143.2 ± 14.8	141.8 ± 16.9	0.650
Platelet (10^9^/L)	197.4 ± 41.4	260.0 ± 59.7	<0.0001^*∗∗∗*^
CRP (mg/L)	1.178 ± 1.56	2.284 ± 6.44	0.012^*∗*^
Albumin (g/L)	44.5 ± 3.13	43.9 ± 3.23	0.307
Urea(BUN) (mmol/L)	5.24 ± 1.46	5.08 ± 1.33	0.578
Potassium (mmol/L)	3.60 ± 0.32	3.57 ± 0.34	0.706
Creatinine (CREA) (*μ* mmol/L)	65.94 ± 18.0	64.51 ± 20.6	0.710
Uric acid(UA) (*μ* mmol/L)	400.7 ± 102.8	335.8 ± 94.5	0.001^*∗∗*^
Blood glucose (mmol/L)	6.57 ± 1.84	6.51 ± 1.41	0.863
Prothrombin time (PT) (second)	18.9 ± 29.9	15.9 ± 23.2	0.578
International standard ratio of prothrombin-(INR)	0.994 ± 0.21	0.957 ± 0.12	0.284
Prothrombin activity (PTA) (%)	105.1 ± 23.7	112.7 ± 17.7	0.072
APTT (s)	40.5 ± 20.6	39.6 ± 12.9	0.800
Fibrinogen(FIB) (g/L)	2.69 ± 1.52	2.85 ± 0.72	0.487
Thrombin time (TT) (second)	23.89 ± 35.5	23.97 ± 37.3	0.992

^#^Swollen degree was estimated by Blaylock classification [24]. ^&^Chronic lung diseases refer to chronic obstructive pulmonary disease (COPD) bronchiectasis mainly; chronic renal failure is diagnosed by physician based on Clinical Practice Guideline for Diabetes and CKD (KDIGO): 2012 update [25]; hepatic dysfunction refer to acute and chronic hepatitis of various etiologies; cardiovascular diseases including coronary heart disease, cor pulmonale; hematologic diseases including hemophilia, anemia.

**Table 5:** Logistic regression analysis of associated factors for PT, PLT, and FIB in 105 T.s patients.

Variables	PT		FIB		PLT	
	OR	*P* values	OR	*P* values	OR	*P* values
Blood glucose	1.64 (0.95–3.12)	0.083	0.92 (0.66–1.30)	0.637	0.89 (0.64–1.21)	0.467
UA	1.15 (1.13–1.19)	<0.0001^*∗∗∗∗*^	0.89 (0.78–0.99)	0.026^*∗*^	0.79 (0.78–0.89)	0.007^*∗∗*^
CREA	0.97 (0.92–1.02)	0.361	1.00 (0.97–1.03)	0.889	1.00 (0.98–1.03)	0.776
Potassium	0.18 (0.01–3.35)	0.261	3.20 (0.78–4.16)	0.109	0.79 (0.19–3.17)	0.747
Albumin	1.14 (0.85–1.60)	0.366	0.84 (0.71–1.00)	0.060	0.92 (0.79–1.09)	0.349
BUN	0.47 (0.19–0.96)	0.064	1.11 (0.77–1.61)	0.562	0.84 (0.58–1.21)	0.366
CRP	0.67 (0.37–1.07)	0.133	1.33 (1.03–1.81)	0.043^*∗*^	1.26 (0.99–1.21)	0.079
Systolic pressure	1.02 (0.98–1.07)	0.320	1.00 (0.97–1.02)	0.960	1.01 (0.99–1.03)	0.445

## Data Availability

Data are available from the corresponding author on reasonable request.
